# Lgr6-expressing functional nail stem-like cells differentiated from human-induced pluripotent stem cells

**DOI:** 10.1371/journal.pone.0303260

**Published:** 2024-05-14

**Authors:** Yukino Inomata, Nano Kawatani, Hiromi Yamashita, Fumiyuki Hattori

**Affiliations:** 1 Innovative Regenerative Medicine, Graduate School of Medicine, Kansai Medical University, Hirakata city, Osaka, Japan; 2 Osaka College of High-Technology, Osaka City, Osaka, Japan; Okayama University: Okayama Daigaku, JAPAN

## Abstract

The nail matrix containing stem cell populations produces nails and may contribute to fingertip regeneration. Nails are important tissues that maintain the functions of the hand and foot for handling objects and locomotion. Tumor chemotherapy impairs nail growth and, in many cases, loses them, although not permanently. In this report, we have achieved the successful differentiation of nail stem (NS)-like cells from human-induced pluripotent stem cells (iPSCs) via digit organoids by stepwise stimulation, tracing the molecular processes involved in limb development. Comprehensive mRNA sequencing analysis revealed that the digit organoid global gene expression profile fits human finger development. The NS-like cells expressed Lgr6 mRNA and protein and produced type-I keratin, KRT17, and type-II keratin, KRT81, which are abundant in nails. Furthermore, we succeeded in producing functional Lgr6-reporter human iPSCs. The reporter iPSC-derived Lgr6-positive cells also produced KRT17 and KRT81 proteins in the percutaneously transplanted region. To the best of our knowledge, this is the first report of NS-like cell differentiation from human iPSCs. Our differentiation method and reporter construct enable the discovery of drugs for nail repair and possibly fingertip-regenerative therapy.

## Introduction

The nail or claw is an important tissue that is evolutionarily conserved in all known land mammals. Human nails are especially important for finger function, sensation, protection, and cosmetic appearance [1]. Inborn malformations, fingertip injuries, and anticancer chemotherapy can lead to intractable abnormalities of the nails [1–5]. Unfortunately, there is no effective treatment for severe cases.

The nail matrix including stem or progenitor cell populations, produces nails throughout life. The nail stem (NS) cells actively divide asymmetrically, and their descendant cells gradually keratinize to form the mature nail. Developmentally, nail rudiments appear on the fingers as epidermal thickenings near the distal end of the dorsal side of the digits approximately 9 weeks after gestation. The nail matrix is formed from the proximal nail fold at approximately 13 weeks [6,7]. Prior to this, the rudiment of the limb, initially called the limb bud, develops from the lateral body wall on day twenty-four. The limb bud has a mesenchymal core and an epithelial cap. The limb bud grows and elongates to form an arm or leg through the leading function of the apical ectodermal ridge (ARE), similar to the blastema in amphibians [8]. ARE secretes fibroblast growth factor-8 and Wnts and stimulates the growth and subsequent segmentation of the arm or leg [9]. Segmentation results in segment-specific expression of homeobox genes. Segmental identifications start from the proximal to the distal. The final distal-most segment expressing Hoxd13 is responsible for digit formation [10].

Interestingly, several groups have reported that the nail matrix can contribute to digit tip regeneration [1,11–13], and this would be dictated by Wnt signaling [11,14]. Leucine-rich repeat-containing G-protein-coupled receptors (Lgrs) are well-known as a stem cell marker protein family in the hair follicle, intestine, and nail [11,15] and may function as an auxiliary receptor in the Wnt pathway to enhance and/or modify the signal [16].

Human pluripotent stem cells (iPSCs) are possible cell sources for various cell replacement therapies. However, the immaturity of human iPSC-derived somatic cells is one of the biggest obstacles. An idea to escape from this—the therapeutic application of somatic stem cells derived from human iPSCs—would be hopeful. Here, we provide the first report describing the differentiation of human iPSCs into NS-like cells expressing the nail stem cell marker Lgr6, for future applications in drug screening and cell administration therapy.

## Materials and methods

### Animals

All animals used in this study were handled according to the Kansai Medical University ethical guidelines for animal experiments. The procedures were approved by the ethical committees (No. 23–35). NOD-SCID mice (CLEA Japan, Inc., Tokyo, Japan) were used for cell transplantation and histological analyses.

### Human-induced pluripotent stem cells (iPSCs)

The hiPSC lines: (RIKEN-2F [17] and 253G1 [18]) were obtained from RIKEN BioResourse Research Center, Ibaraki, Japan.

### Maintenance and digit organoid differentiation of human iPSCs

The human iPSCs were differentiated into limb bud organoids with reference to the previously reported methods with some modifications [19]. In this report, differentiation experiments for data acquisition were performed using the 253G1 and RIKEN2F cell lines. Both cell lines differentiated into similar digit organoids. Undifferentiated human iPSCs were maintained on plastic dishes (Corning Inc., NY, USA) coated with 0.5 μg/cm^2^ iMatrix511 Silk (Nippi Inc., Tokyo, Japan) using StemFit AK02N (Ajinomoto, Tokyo, Japan) as the culture medium. The dispersed cells were seeded at a density of 15,000 cells/cm^2^ with a 10 μM Rho kinase inhibitor, Y-27632 (Selleck Biotech, Kanagawa, Japan). One day later, the medium was changed to the same medium without Y-27632. Passage of the cells was performed every three to four days. The cells were detached from the culture dish by treatment with TrypLE express enzyme (Thermo Fisher Scientific, Waltham, MA, USA) supplemented with 10 μM Y-27632 for 20 min at 37°C. For differentiation, five days before the initiation of differentiation (Day 0), the single cells were dispersed into StemFit AK02N with a 10 μM Y-27632 and distributed to a noncell adhesive 96 well plate (PrimeSurface 96U, Sumitomo Bakelite, Tokyo, Japan) or a sphere dish (10 cm EZ sphere having 17,000 wells, AGC TECHNO GLASS Co., Ltd., Shizuoka, Japan) at a density of 3500 cells per well with 200 μL / well for 96 well plate, or 500 cells per well with 30 mL for EZ sphere. For 96 well plate, one day before Day 0, aspirate 100 μL of medium from all wells and add 100 μL of Essential 8 (E8) medium (Thermo Fisher Scientific) with a 10 μM Y-27632. For EZ sphere, add 10 mL of E8 medium. For 96 well plate, on Day 0, half of the medium was exchanged with Essential 6 (E6) medium (Thermo Fisher Scientific) containing 20 ng/ml bone morphologic protein 4 (BMP4). For EZ sphere, on Day 0, all formed cell aggregates were collected into a tube, then left to sit for three minutes for natural sedimentation. Half of the medium was exchanged with Essential 6 (E6) medium (Thermo Fisher Scientific) containing 20 ng/ml BMP4. The cells were transferred to four cell-non adhesive 10-cm dishes (Cell-Repellent Surface, Greiner Bio-One Kremsmünster, Austria). On differentiation days 2, 3, and 6, each half of the medium was aspirated, and each the same amount of E6 medium with 10 ng/ml BMP4 was supplied. On differentiation Day 8, each half of the medium was aspirated, and each the same amount of Dulbecco’s modified Eagle medium (DMEM) (Fujifilm Wako Chemical Inc., Osaka, Japan) supplemented with a 1x concentration of insulin, transferrin, and selenium mixture (ITS, Thermo Fisher Scientific) and 10 ng/ml BMP4 was added. On Day 9, half of the culture medium was changed with DMEM supplemented with 1x ITS, 10 ng/ml BMP4, and 100 μM all-trans retinoic acid (RA). On Day 10, half of the culture medium was changed with DMEM supplemented with 1x ITS, 10 ng/ml BMP4, and 2 μM LDN193189 (Selleck Biochemical). For 96 well plate, on Day 13, all spheres were collected into a centrifugation tube using a 1000 μL wide-bore chip (Axygen Corning Inc.) and centrifuge at 120 g for 5 minutes. They were transferred to a cell-non adhesive 10-cm dish (Greiner Bio-One) with 20 mL of organoid maturation medium (OMM) which is consisted with 1:1 mixture of DMEM/F12 (Fujifilm Wako Chemical) and Neurobasal medium (Thermo Fisher Scientific), supplemented with 0.1 mM non-essential amino acids, 1 mM glutamine, 55 μM 2-mercaptoethanol, 0.5% N-2 supplement (Thermo Fisher Scientific), 0.5% B-27 supplement (Thermo Fisher Scientific), 50 μg/mL ascorbic acid 2-phosphate (Merck KGaA, Darmstadt, Germany), 0.05% bovine serum albumin (Merck) supplemented with 7 μM CHIR-98014 (Selleck Biotech), 10 ng/mL FGF-basic (Nacalai Tesque Inc., Kyoto, Japan), 10 ng/ml FGF8 (Peprotech, Thermo Fisher Scientific), and 4 μg/ml hydrocortisone (Tokyo Chemical Industry Co., Ltd., Tokyo, Japan), and started gyratory rotation culture with approximately 60 rpm of agitation-speed until the end of the culture experiment with changing medium every third day. For EZ sphere, on Day 13, all spheres were collected into centrifugation tubes and centrifuged at 120 g for 5 minutes. The collected spheres were transferred into eight cell-non adhesive 10-cm dishes (Greiner Bio-One) with each 20 mL of OMM, and started gyratory rotation culture with approximately 60 rpm of agitation-speed until the end of the culture experiment with changing medium every third day.

### Lgr6-GFP reporter iPS development

We chose the tentative promoter region as the 1 kb of upstream sequence from the first codon of Lgr6 variant 2 (GenBank: AL356953.17. from 19771 to 20838). The Kozak sequence (GCCACC) was inserted just above the first codon in EGFP. The piggyback-based reporter vector has 5’ and 3’ inverted terminal repeat sequences (ITRs), and between them, it has Lgr6 promoter-Kozak-EGFP-rabbit beta globin (rBG) polyA signal and cytomegalovirus promoter (CMV)-puromycin-resistant gene-bovine growth factor (BGH) polyA signal sequences. The designed vector was constructed and purchased from VectorBuilder (VectorBuilder Inc., Kanagawa, Japan). We transduced the construct into 253G1 and RIKEN2F cell lines using a piggyback transposase expression vector (System Biosciences, LLC., CA, USA). Three to four days later, the gene-transduced cells were selected by two to three days of treatment with 2 μg/ml puromycin (Thermo Fisher Scientific) and subsequently cultured to form single cell-derived colonies for several days. We manually selected individual colonies and further expanded them for functional tests. We performed small-scale digit organoid differentiation and successfully selected functional clones using fluorescent microscopy.

### Global gene expression profile of digit organoid in comparison with undifferentiated human iPSCs

Total RNA was extracted from the cells using ISOGEN (NIPPON GENE CO., LTD., Tokyo, Japan) according to the manufacturer’s instructions. Quantification, quality check of the total RNA and library development, and sequencing were performed by AZENTA LIFE SCIENCES, Massachusetts, USA. The total RNA was quantified and qualified by NanoDrop, Qubit RNA Assay (Thermo Fisher Scientific), and TapeStation RNA ScreenTape (Agilent Technologies, California, USA). Total RNA samples (109 ng) that met the quality guideline RIN: 8 or higher were treated with the NEBNext Poly(A) mRNA Magnetic Isolation Module (New England Biolabs, Massachusetts, USA) to enrich poly-A mRNA and remove rRNA molecules. cDNA synthesis followed by transcriptome library preparation was conducted using the NEBNext Ultra II Directional RNA Library Prep Kit for Illumina, where dUTP was incorporated in the process of second-strand cDNA synthesis instead of dTTP, which blocks PCR amplification against the 2nd strand templates. This enables us to maintain the strandness of RNA transcripts. A 13-cycle PCR amplification was performed to increase the library yield and to incorporate sample barcodes into the library fragments. The resulting transcriptome libraries were quantified using the Qubit DNA Assay (Thermo Fisher Scientific), and their fragment size distribution was estimated using TapeStation D1000 ScreenTape (Agilent Technologies). The libraries were loaded onto a next-generation sequencing platform, NovaSeq 6000 (Illumina, Inc., California, USA). Sequencing was performed according to the manufacturer’s instructions with a 150-bp paired-end configuration, yielding approximately 6 GB per sample. Raw reads with low quality and adapter sequences were removed using Cutadapt v.2.9. The remaining reads were then mapped to the human genome (GRCh38 version 101) by HISAT2 v.2.2.0 (PMID: 25751142), and gene expression was quantified using the R package featureCounts v.2.6.0 (PMID: 24227677). Consequently, differentially expressed genes (DEGs) were identified on the basis of differences in expression levels (log2 fold-change > 1 and adjusted pvalue < 0.05) between samples after removing genes with zero read counts using DESeq2 v.1.6.3 (PMID: 25516281). Gene Ontology (GO) and pathway enrichment analyses were carried out with SRplot [20].

### Quantitative polymerase chain reaction (qPCR) analysis

Total RNA was extracted from the cells using ISOGEN according to the manufacturer’s instructions. Reverse transcription of 50 ng of RNA was performed using SuperScript^®^ Reverse Transcriptase (Thermo Fisher Scientific) with an oligo(dT)20 primer. To investigate various gene expressions, a quantitative polymerase chain reaction was performed using GeneAce SYBR^®^ qPCR Mix α (NIPPON GENE CO., LTD.) with gene-specific primer sets listed in S1 Table. The expression levels of other genes were also investigated. All gene expression levels were normalized to the internal ribosomal protein S18 expression levels.

### Immunofluorescent staining

Organoids were fixed in 4% paraformaldehyde for 20 min at 25°C. The organoids were then washed twice with Tris-buffered saline containing 0.2% Tween-20 (TBS-T) and incubated with 0.1% Triton-X100 containing TBS-T for 10 min at 25°C, followed by immersion in 30% sucrose containing TBS-T for over a night at 4°C. Organoids were settled on the bottom of the cup (Cryomold^®^ No. 1, Sakura Finetek Japan Co., Ltd., Tokyo, Japan), and the solution was aspirated completely by paper absorption (Kimwipe^®^, Nippon Paper Crecia Co., Ltd., Tokyo, Japan) from the ping hole made by the tip of a 29G needle. Subsequently, the cup was filled with O.C.T. compound (Sakura Finetek Japan Co., Ltd.) and cooled to -80°C for making cryoblocks. The cryoblocks were applied to 8 μm thick sectioning and adhered to aminosilane-treated slide grass (Matsunami Glass IND., Ltd., Osaka, Japan). After being well dried, the slide grasses were dipped in TBS-T to wash away the residual O.C.T. compound and rehydrate the organoid. The slides were treated with a blocking solution (Nacalai Tesque) for 30 minutes at 25°C. The first antibody-containing blocking agent was applied and incubated overnight at 4°C with paraffin sealing to prevent evaporation. The cells were then washed three times with TBS-T and immersed in the second antibody-containing blocking agent for 1 h at 25°C. After washing three times, fluorescent signals were observed using a fluorescence microscope (Eclipse Ti2, Nikon Instruments, Tokyo, Japan) controlled by the equipped software (NIS-Elements, Nikon Instruments). The primary and secondary antibodies used are listed in S2 Table. Nuclear DNA were stained by 4’,6-diamidino-2-phenylindole (DAPI) (Thermo Fisher Scientific).

### Enrichment of Lgr6-GFP reporter-expressing cells by fluorescent activated cell sorting (FACS)

Approximately one hundred digit-organoids (day 62) that were differentiated from Lgr6-GFP reporter-iPSCs were collected in a 50-ml centrifuge tube and washed once with 30-ml of Hanks buffer (Nacalai Tesque). Then the medium was completely changed by the Hanks buffer containing 0.1% collagenase (Fuji Film Wako Chemical Inc.). A micromagnetic stirrer bar (AS ONE Corporation, Osaka, Japan, MC-52) was dropped in the tube and the enzymatic solution was mixed using a magnetic stirrer (AS ONE, CB-4) with maximum speed for 1 hour at 37°C. After confirmation of the complete dispersion under a conventional microscope, the cells were collected and washed once with Hanks buffer. The dispersed cells were passed through the mesh of a 40 μm pore (Becton, Dickinson and Company (BD), New Jersey, USA, Cell Strainer 40 μm Nylon). The cell suspension solutions were kept cold on ice until the sorting experiment. We used a FACS machine (BD, FACS Aria III) according to the manufacturer’s instructions. After doublet elimination by the height and width subparameters in the forward scatter and the side scatter, we determined the medium- to strong-GFP-expressing cells in the histogram for sorting.

### Production of the cell sphere and subcutaneous cell transplantation

The sorted 3.4 x 10^6^ cells were seeded in a sphere plate (AGC, EZ-Sphere plate 100mm) for 200 cells/well with OMM medium and incubated for four days [21]. The produced cell-spheres were collected by centrifugation and then subcutaneously injected using a 29-gauge tuberculin syringe (NIPRO CORPORATION, Osaka, Japan) under the shaved back skin of immunodeficient NOD-SCID mice (CLEA). After 20 days of transplantation, the hard lump tissue formed under the skin was removed.

### Statistical analysis

Statistical analyses were performed using EZR software (Jichi Medical University, Japan) [22]. For comparisons with differentiation day 0, significant differences were determined using one-way analysis of variance (one-way ANOVA), followed by post hoc testing using the Dunnett test vs. undifferentiated hiPSCs. Statistical significance was set at p < 0.05.

## Results

### Differentiation of digit organoids via the extended culture of limb bud organoids with gyratory rotation

**Fig 1:**
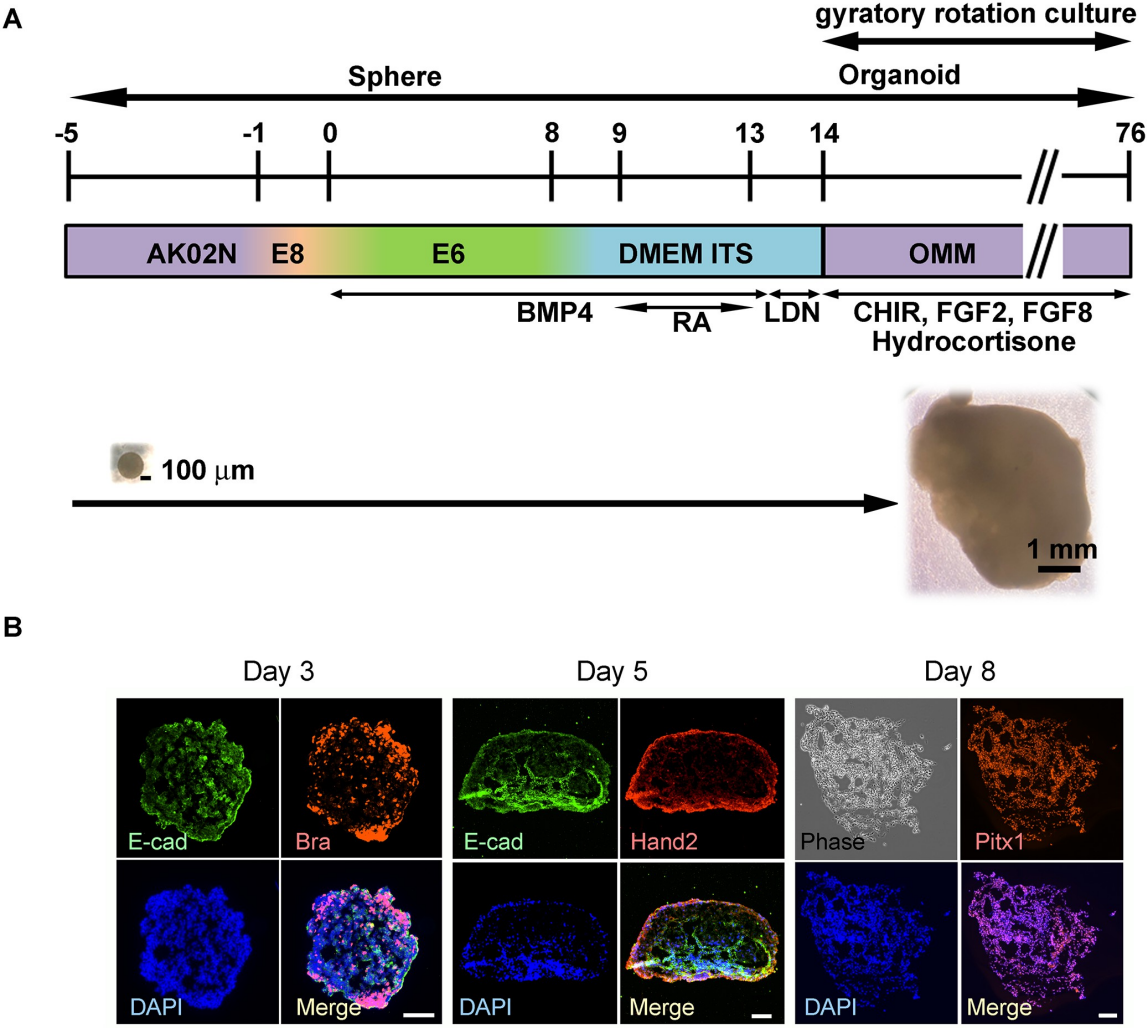
Differentiation scheme of digit organoid and early expressions of limb bud-related proteins. (A) Differentiation scheme; (B) Immunofluorescent staining of early differentiating spheres regarding E-cadherin, T-brachyury, Hand2, and Pitx1. The used antibodies are shown in the S2 Table. Scale bars = 200 μm.

To achieve differentiation of digit appendix nail tissue from human-induced pluripotent stem cells (iPSCs), we referred to a previous report describing limb bud differentiation from murine iPSCs and modified and extended their method [19]. We sequentially treated spheres of iPSCs with a floating culture of the limb bud organoids. Here, we adopted a gyratory rotation culture for further growth and differentiation of the organoids (Fig 1A). Sequential expression of E-cadherin, brachyury, Hand2, and Pitx1 confirmed the earlier successful differentiation of the limb bud organoids (Fig 1B). The results confirmed successful reconstitution of limb bud differentiation.

### mRNA expression of segment patterning and digit-related genes during gyratory rotation culture

**Fig 2:**
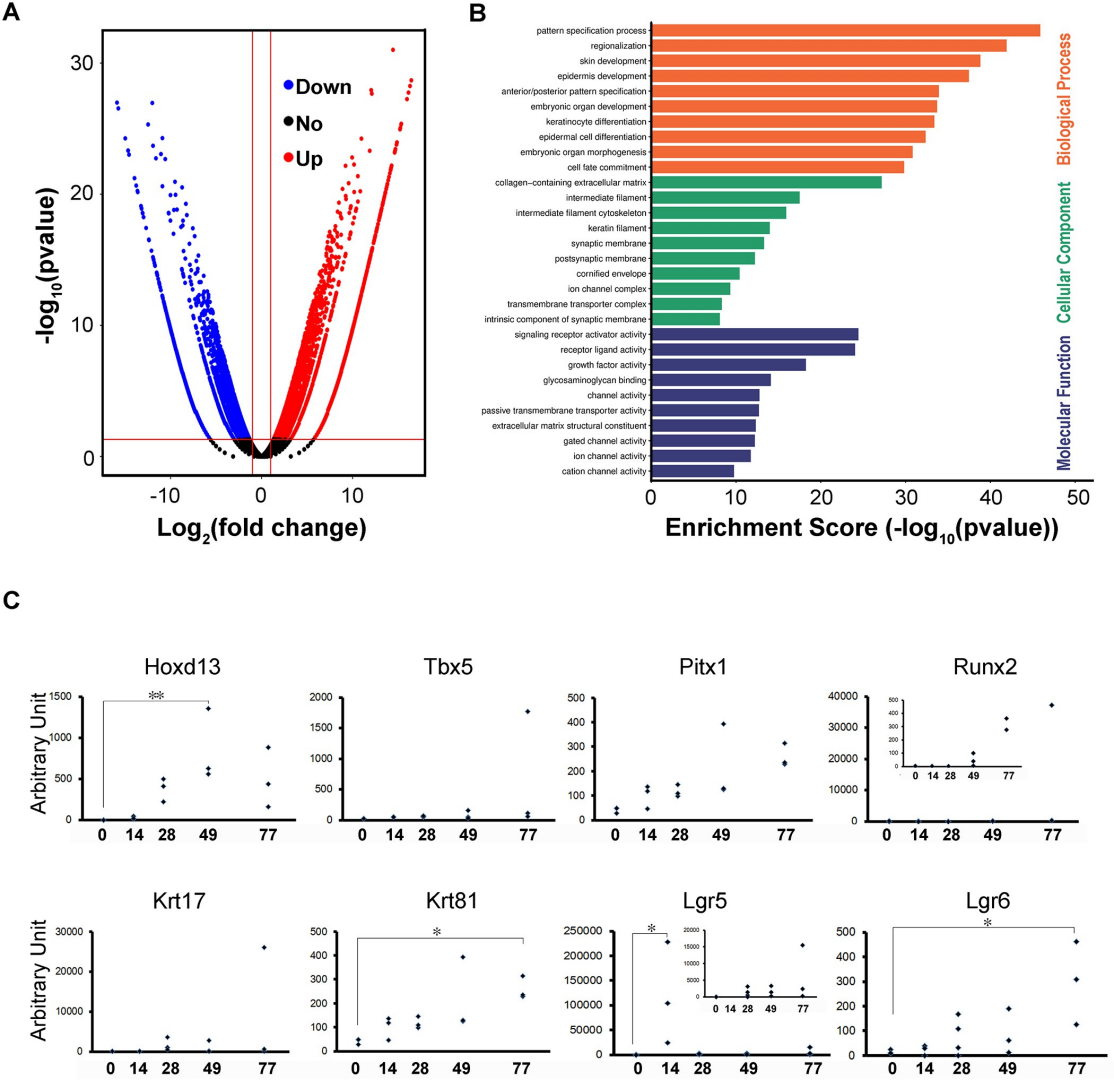
Global gene expression profiling by RNA sequencing and time course of the gene expressions from pluripotent to digit organoid via limb bud formation. (A) Volcano plot of genes expressed in D0 undifferentiated iPSCs vs. D50 digit organoids. (B) GO enrichment analysis of differently expressed 3845 genes (|log2 (fold change)| >2.5) with statistical significance. The log10 (pvalue) scores of GO terms are in three ontologies: biological process (GO:0008150), cellular component (GO:0005575), and molecular function (GO:0003674). (C) Time course of mRNA expression levels of the genes of interest by qRT-PCR analysis. Gene expression levels were normalized to internal ribosomal protein S18 expression levels. Statistical analysis was done using the Dunnett test vs. undifferentiated hiPSCs. The significance is **p < 0.01, *p < 0.05. Data are shown as mean ± SD.

We attempted to achieve long-term culture of the organoids; however, we found that larger grown organoids had necrotic cores in static floating culture. Therefore, we maintained a continuous medium flow during the extended culture period. Gyratory rotation culture was performed for further growth and differentiation of the organoids. First, we investigated the global mRNA expression pattern of the digit organoid on differentiation day 50 compared with that of the undifferentiated hiPSCs. We found both upregulated (4744) and downregulated (4280) genes (Fig 2A). Gene Ontology (GO) enrichment analysis of differentially expressed 3845 genes (|log2 (fold change)| >2.5) suggested that they link to various GO terms in three major GO categories: biological process, cellular components, and molecular function (Fig 2B) (S3–S5 Figs). Among them, biological process contains the largest number of 2723 genes, and those terms were linked to development (S3 Fig). One of the GO terms in cellular components suggests active extracellular matrix production in the digit organoid (S4 Fig). Several GO terms in molecular function suggested that there were rich receptor-ligand interactions and cross-membrane activities in the digit organoid (S5 Fig). Furthermore, pathway enrichment analysis suggested that gene expression alterations along with differentiation would enhance the canonical Wnt signaling pathway (S6 and S7 Figs). We selected the genes that have been previously reported in relation to limb and digit development from differentially expressed genes for transcriptional regulating factors, morphological factors, and keratins and showed them in S1 and S2 Figs. Pluripotent stem cell-specific transcriptional regulatory factors, including ZSCAN10, LINC00678, VRTN, NANOG, LIN28A, POU5F1, LCK, ESRG, and IDO1, were downregulated with differentiation [23]. In turn, homeobox proteins including HOXD13 [23,24]; Lgr5 and 6 [11]; T-box genes [25]; DLX-genes [26]; Pitx1 and 2 [27,28]; Eph receptors [29]; SOX-genes [30]; LIMX1A and B [31]; RUNX1, 2, 3 [32]; and Homeobox protein engrailed-1 [33] were upregulated with differentiation. The gene expression levels of morphological factors, including BMPs [9], GFD11 [34], fibroblast growth factors, including FGF8 [35], WNTs and WLS [36], GREM1, 2, and SHH [37], increased with differentiation. Nail-related hard keratin genes, including KRT6A, B, C, KRT16, KRT17, and KRT31 [38,39], were highly expressed in the digit organoids. Next, we investigated the time course of the expression levels of various key gene mRNAs regarding arm segmentation, skeletal bone formation, nail matrix differentiation, and nail formation by quantitative PCR (Fig 2C). Hoxd13, which is a transcription factor responsible for digit formation and whose gene expression levels peaked at differentiation day 49, shows successful differentiation of the most proximal digit segment. Pitx1 was continuously expressed in differentiated mesenchymal cells. Runx2 expression was elevated on differentiation day 49 and prominently expressed on day 77. The Runx2 protein is responsible for skeletal bone formation, supporting further advancement of the differentiation process into the bone formation phase. Lgr5-expressing cells were reported to exist in the hair follicle and dorsally to the nail in the proximal fold [11]. We observed that Lgr5 expression was keenly elevated on differentiation day 14. However, it showed a rapidly decreasing trend, and then showed a gradual increase again with the differentiation days. In contrast, Lgr6 expression gradually and continuously increased with differentiation. The expression of typical nail keratin, KRT17 (type I; KRT17 is also known as CK-17, PCHC1) [40] and KRT81 (type II; also known as hHb1, MLN137, ghHkb1, hHAKB2-1) [41], gradually increased with differentiation.

### Neighboring protein expressions of Lgr6, KRT17, and KRT81 in digit organoids

**Fig 3:**
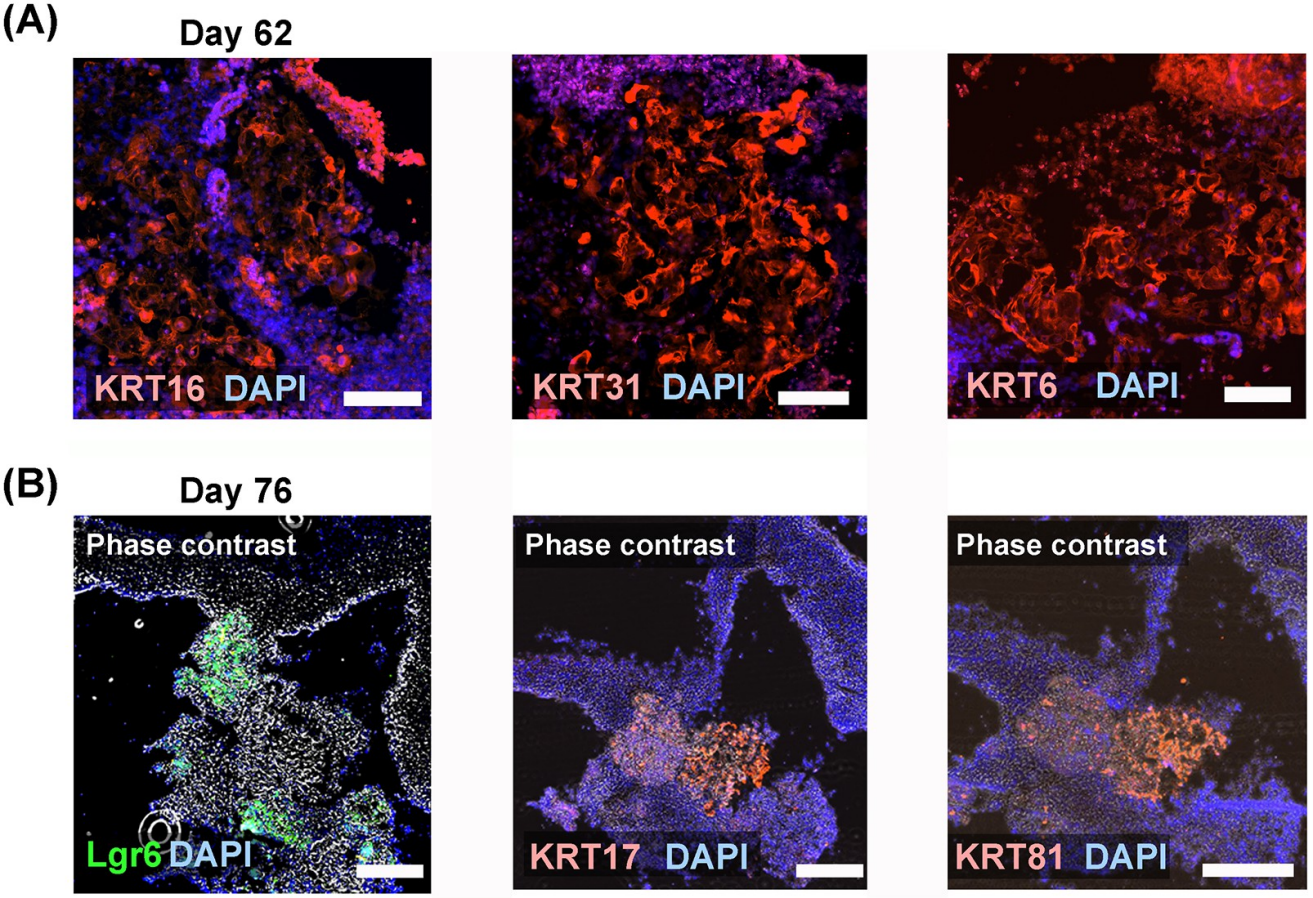
Immunohistochemical detection of Lgr6 and hard keratins. (A) Immunohistochemical staining of a differentiation day 62 organoid detected the protein expression of type 1 keratins (KRT16 and KRT31) and a type 2 keratin (KRT6). Scale bars = 100 mm. (B) Immunohistochemical staining of a differentiation day 76 organoid detected Lgr6, type 1 keratin: KRT17, and type 2 keratin: KRT81 protein expressions in the continuous sections. Scale bars = 200 μm. The used antibodies are shown in the S2 Table.

Immunohistochemical staining for the day 62 digit organoid revealed that the protein expression of type 1 keratins (KRT16 and KRT31) and a type 2 keratin (KRT6) was expressed in the enucleating cell clusters (Fig 3A). Furthermore, we demonstrated a continuous section of a day 76 organoid and confirmed Lgr6, KRT17, and KRT81 protein expressions in neighboring locations (Fig 3B).

### Development of a functional human Lgr6-GFP reporter cell line, enrichment of reporter positive cells, and transplantation

**Fig 4:**
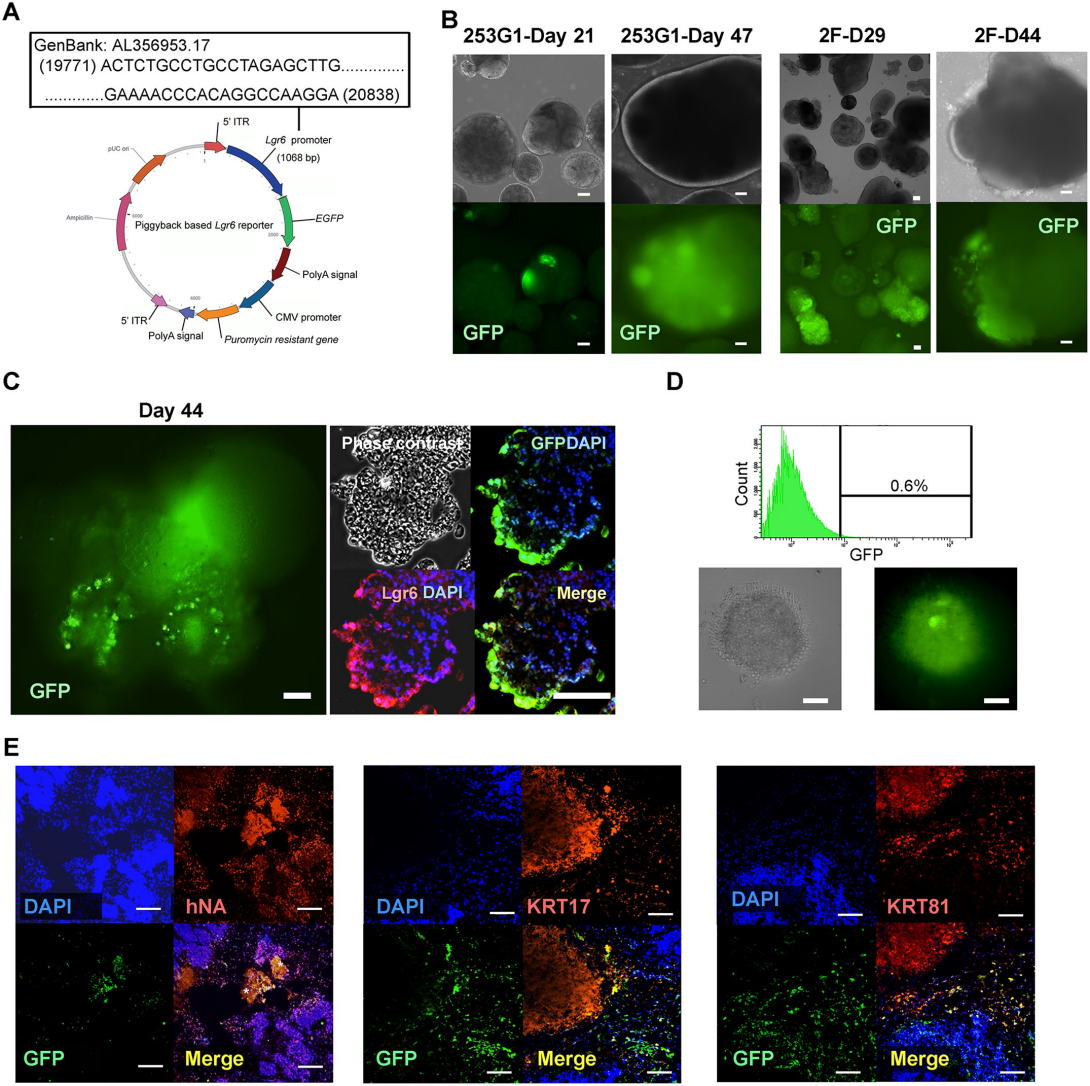
Functionality of the Lgr6-GFP reporter iPSC. (A) Design of the Lgr6-GFP reporter gene on the piggyback system (B) Expression of Lgr6 reporter GFP expression on day 30 of the organoids produced by EZ sphere dishes. Scale bar = 100 μm. (C) Expression of a reporter GFP-expressing organoid produced by a 96 well-plate at the differentiation day 44. Immunohistochemical detection of Lgr6 protein is in red. Scale bar = 100 μm. (D) Cell sorting of GFP-positive cells from day 62 organoids. The enriched Lgr6-GFP-positive cells were formed into spheres. Scale bar = 100 μm. Immunofluorescent staining of the isolated lump tissue after 20 days of the subcutaneous injection into the immunodeficient mouse for (E left) GFP and human nuclear antigen (hNA), scale bar = 200 μm, (E middle) GFP and KRT17, scale bar = 100 μm, and (E right) GFP and KRT81, scale bar = 100 μm. The asterisks in the left panel indicate parts of red-colored autofluorescence. The used antibodies are shown in the S2 Table.

We searched the Eukaryotic Promoter Database (EPD) [42] and identified three Lgr6 alternative splicing variants. Because the most obvious signals were shown in variant 2 by Cap Analysis of Gene Expression (CAGE) analysis of FANTOM5 [43] and ENCODE [44], we chose approximately 1 kb of upstream sequence from the first codon of variant 2 for the Lgr6 reporter construct (Fig 4A). We established several clones of the gene transduced into human iPSC and demonstrated digit organoids formation in parallel for screening. Using the best reporter iPSC-clone, we found that approximately 10 to 30% of the organoids indicated green fluorescence signals and the positive ratio was slightly varied by the differentiation period (Fig 4B). We performed immunofluorescent stains for GFP and Lgr6 on the cryosection digit organoid. We found co-localized expression of reporter-GFP and Lgr6 proteins (Fig 4C). We then enriched the GFP-expressing cells from organoids (day 62) using cell sorter (Fig 4D) and formed 17,000 spheres, each containing 200 cells. After four days, we performed a subcutaneous injection under the shaved back skin. Twenty days after transplantation, we found relatively hard lump tissue by palpation. The tissue was suggested to be composed of host and graft-derived chimeric regions (S8A Fig). The engrafted human cell-rich regions contained the host-derived vascular endothelial cells and the host red blood cells (S8B and S8C Fig). A part of We immunohistochemically investigated it and found the human nuclear antigen- and reporter GFP-double positive cells (Fig 4E left), and nearby there was a denuclearized KRT17- and KRT81-double-positive area (Fig 4E middle and right).

## Discussion

To achieve differentiation of nail stem (NS)-like cells from human-induced pluripotent stem cells (iPSCs), we followed a previously reported method for limb bud formation using murine iPSCs for 10 days [19]. We confirmed the apical ectodermal ridge-like cell formation by the expression of E-cadherin. Hand2 expression suggested limb mesenchymal cell development. Both forelimb- and hindlimb-specific expressing genes, Tbx5 and Pitx1 were observed, respectively, which shows both forelimb and hindlimb buds were differentiated in our experiments.

To advance the differentiation of limb bud organoids, we tested static, vertical, and horizontal rotation culture systems. As a result, we constantly found the largest organoids in horizontal gyratory rotation culture through repeated differentiation batches. Therefore, we adopted the gyratory rotation culture system for further experiments. Our gyratory cultivation system may enhance the differentiation of larger organoids through the efficient exchange of air and nutrients with excreted waste under less shear stress. As a result, in comparison with the previous report, organoids could express the skeletal bone formation marker Runx2 mRNA without fetal transplantation.

We demonstrated Lgr6 mRNA and protein expression with approximately 2 months of differentiation, suggesting successful differentiation of NS-like cells from human iPSCs. To further confirm the nail-forming ability of NS-like cells, we performed immunohistochemical staining for the nail-enriched type I keratin KRT17 and type II keratin KRT81 and found that KRT17 and KRT81 co-localizing areas exist near the Lgr6-expressing cells, strongly suggesting that human iPSC-derived NS-like cells may have the ability to produce nail plates.

Type I keratin KRT17 and type II keratin KRT81 are known as "hard keratin" because they form hair and nails by making heterotypic complexes. Lgr6- or Lgr5-positive cells are also known to be stem cell populations in hair follicles [45,46]. It is difficult to completely distinguish hair, nails, and their stem cells just from their molecular signatures. We consider two reasons why we think our differentiated cells are NS-like cells. First, we traced the developmental process toward nail formation. We observed a time-dependent elevation of the digit-specific Hoxd13. Fetuses normally have no active hair (lanugo) follicles in their arms or digits [47]. Furthermore, the immunohistochemical positivity showed that a relatively large area did not have any features of a hair follicle, strongly supporting our hypothesis that differentiated tissues in digit organoids should be a combination of NS-like cells and nails. The second is the major difference in comparison with the previous publication describing hair and hair follicles involving organoids derived from human-embryonic stem cells [48]. Lee et al. reported hair-bearing human skin produced by long-term culture of skin organoids. They claimed that their skin organoids were equivalent to human facial skin. Noteworthy, their single-cell mRNA expression analysis revealed that their organoids consisted of only ectodermal cells without any endoderm or mesoderm population. Our digit organoid apparently involves mesodermal cells. The initial sphere richly involved brachyury-positive mesodermal mesenchymal cells. In the latter phase of differentiation, Runx2 mRNA-expressing cells, which might be osteoblasts that form skeletal bones [49], have emerged. From the above contexts, we concluded that our differentiated digit organoids contain NS-like cells and primitive nails.

We demonstrated enrichment of Lgr6-expressing NS-like cells by Lgr6-GFP reporter expression from the digit organoid and subcutaneously transplanted them after sphere formation. The engrafted human cell-derived tissue was connected to the host-derived tissue and received the host capillary vessels and blood. The functionality of the Lgr6-GFP reporter in human iPSC lines was supported by immunofluorescent detection of hard keratin deposition near the Lgr6-expressing cells. Our future goal is to utilize purified NS-like cells for drug screening for nail growth deficiency and cell administration therapy for patients with congenital anonychia and hyponychia patients.

## Conclusions

We provide the first report describing the differentiation of functional nail stem-like cells from human-induced pluripotent stem cells via limb bud organoids by step-wise stimulations tracing molecular processes in limb and digit development. We also established functional Lgr6-reporter human iPSCs.

## Supporting information

S1 Checklist (DOCX)

S1 Fig Gene expression changes (log fold change) in the comparison between differentiation day 50 digit organoid vs. unfdifferentiated hiPSCs. (PDF)

S2 Fig Fragments per kilobase of exon per million reads mapped (FPKM) in D0 (undifferentiated hiPSCs) and differentiation day 50. Transcriptional regulatory factors (A), Morphological factors (B), and Keratins (C). The color strength of each column was assigned by the maximum-to-minimum numbers in each gene category.
(PDF)

S3 Fig The GO enrichment analysis of biological process (GO:0008150). The x-axis shows the enrichment score (-log10(pvalue)). The differentially expressed gene numbers in each term (gene group) are shown as the size of the bubbles. Each pvalue is also shown in color, as shown in the figure.
(PDF)

S4 Fig The GO enrichment analysis of cellular component (GO:0005575). The x-axis shows the enrichment score (-log10(pvalue)). The differentially expressed gene numbers in each term (gene group) are shown as the size of the bubbles. Each pvalue is also shown in color, as shown in the figure.
(PDF)

S5 Fig The GO enrichment analysis of molecular function (GO:0003674). The x-axis shows the enrichment score (-log10(pvalue)). The differentially expressed gene numbers in each term (gene group) are shown as the size of the bubbles. Each pvalue is also shown in color, as shown in the figure.
(PDF)

S6 Fig The pathway enrichment analysis of differently expressed 3845 genes. The gene expression changes were shown as the color of the gene-name box, as shown in the figure.
(PDF)

S7 Fig The pathway view of Wnt signaling pathway in S6 Fig. The gene expression changes were shown as the color of the gene-name box, as shown in the figure.
(PDF)

S8 Fig (A) Immunofluorescent staining focusing on the border zone between host- and the transplanted human-derived tissues in the lump tissue for mouse CD31 (red), human nuclear antigen (green) and all nuclear staining with DAPI (Blue). Scale bar = 20 μm (B) Immunofluorescent staining of the lump tissue-derived cryo-sections for mouse CD31 (red), human nuclear antigen (green). The right panel indicates the zoomed images of the white square-indicated area in the merged image. Scale bar = 50 μm (left), 10 μm (right). (C) Immunofluorescent staining for TER-119 (red) and human nuclear antigen (green). The right panel indicates the zoomed images of the white square-indicated area in the merged image. Scale bar = 50 μm (left), 10 μm (right). The used antibodies are shown in the S2 Table.
(PDF)

S1 Table Primer sequences. (PDF)

S2 Table First and second antibodies for immunofluorescent staining. (PDF)
