# Supplementary figures and images for: Lgr6-expressing functional nail stem-like cells differentiated from human-induced pluripotent stem cells
Source: PLoS One. 2024 May 14;19(5):e0303260. doi: 10.1371/journal.pone.0303260 (PMC11093308; doi:10.1371/journal.pone.0303260)

S1 Fig

Gene Expression changes D50/D0

(Log fold change)

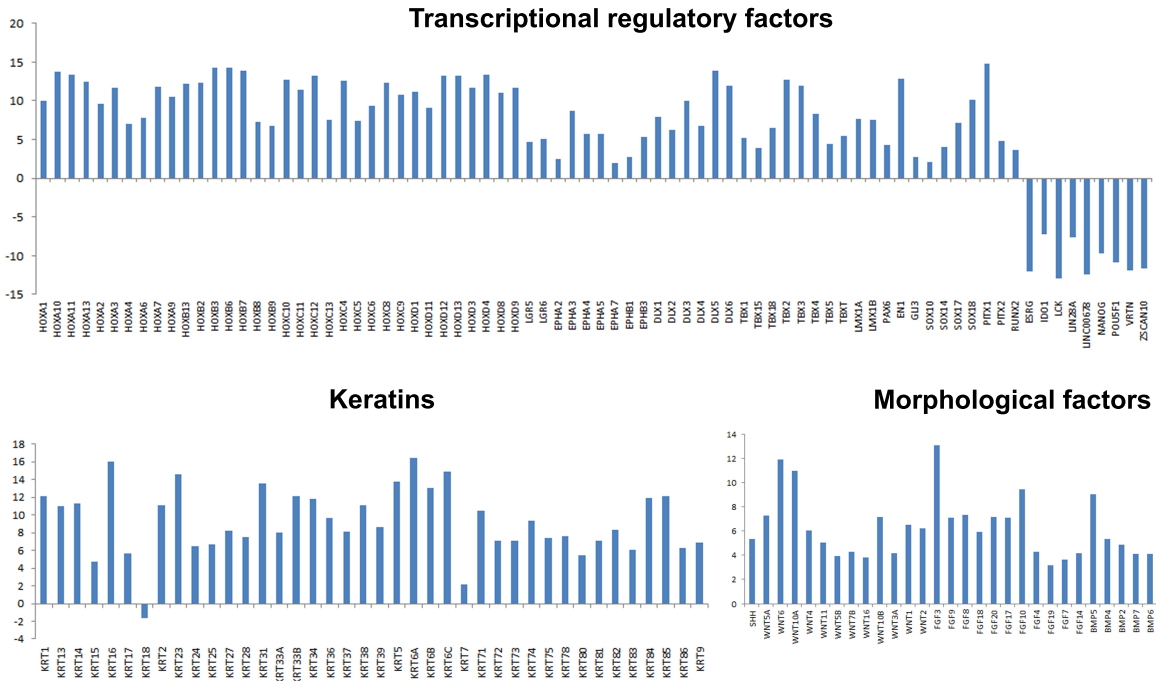

Supplement: S1 Fig — (PDF) [file pone.0303260.s002.pdf]

S3 Fig

Biological Process

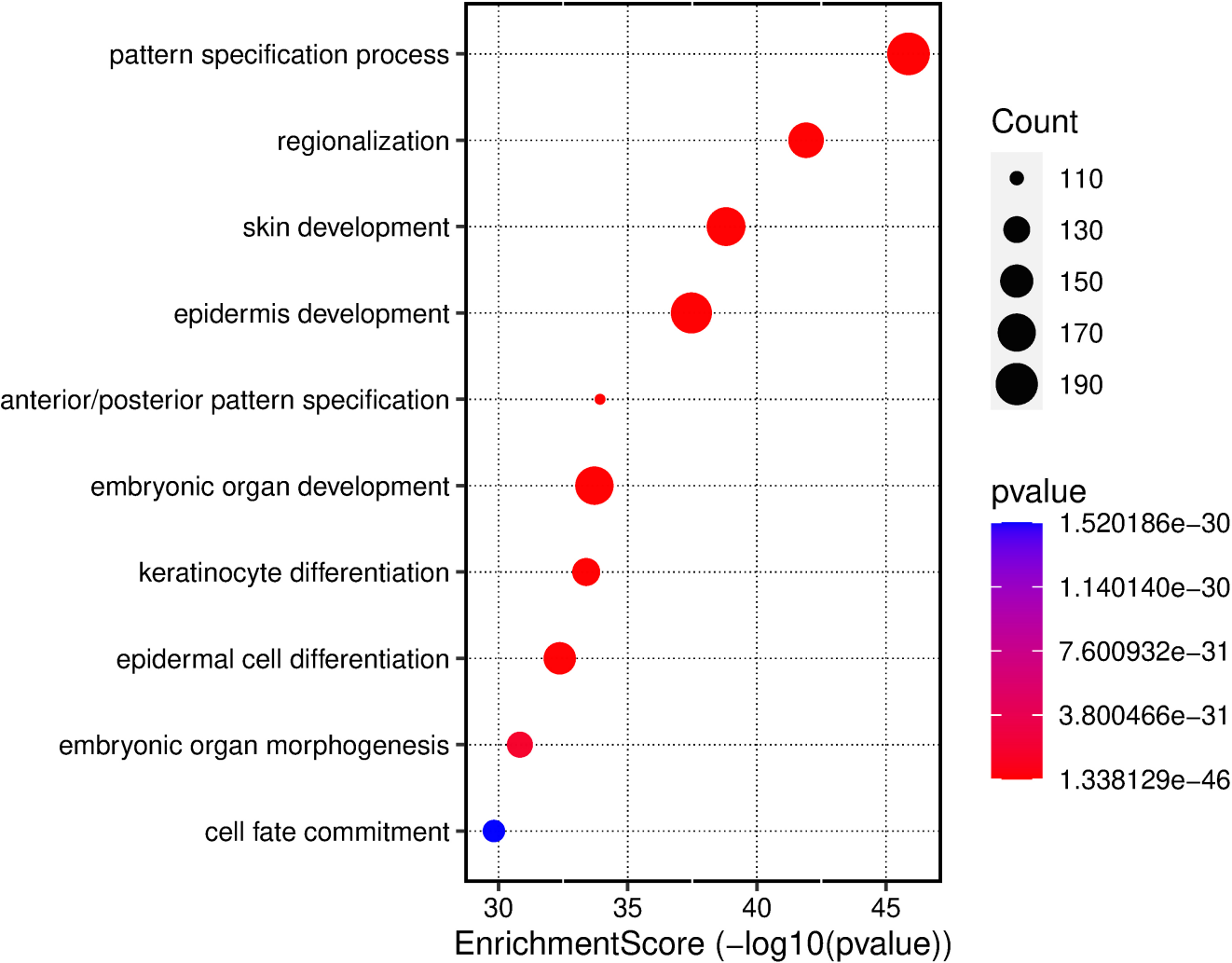

Supplement: S3 Fig — The x-axis shows the enrichment score (-log10(pvalue)). The differentially expressed gene numbers in each term (gene group) are shown as the size of the bubbles. Each pvalue is also shown in color, as shown in the figure. (PDF) [file pone.0303260.s004.pdf]

S4 Fig

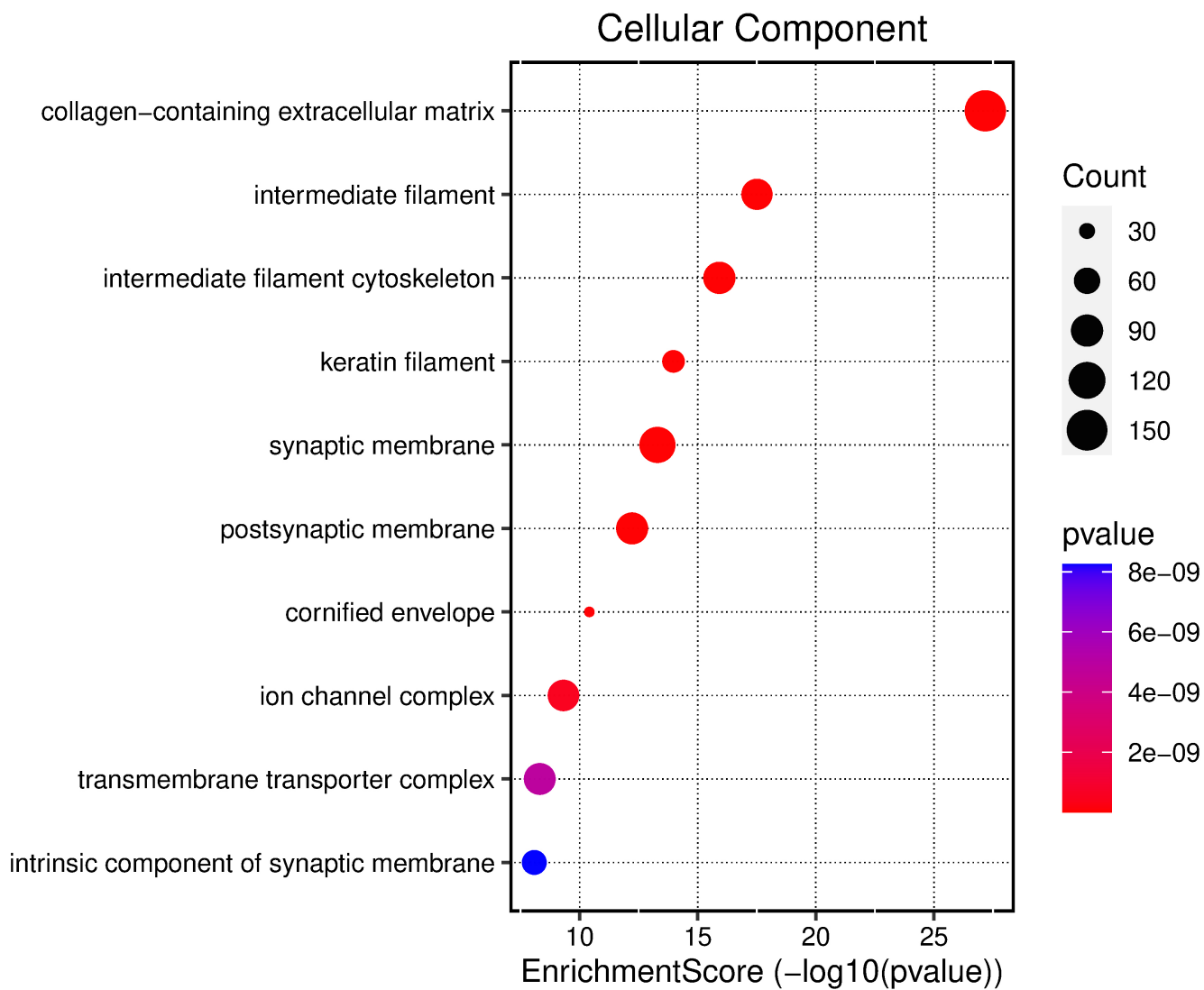

Supplement: S4 Fig — The x-axis shows the enrichment score (-log10(pvalue)). The differentially expressed gene numbers in each term (gene group) are shown as the size of the bubbles. Each pvalue is also shown in color, as shown in the figure. (PDF) [file pone.0303260.s005.pdf]

S5 Fig

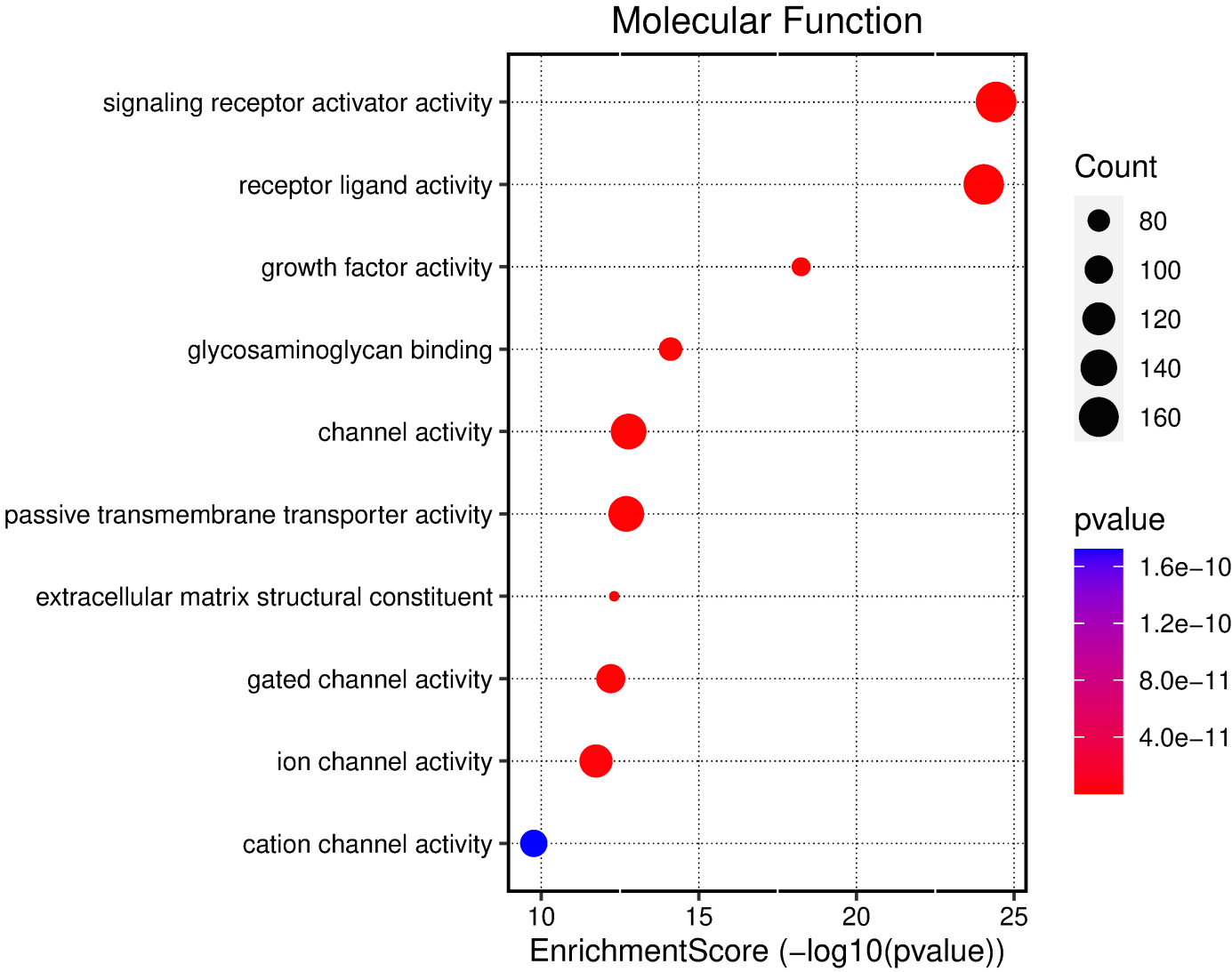

Supplement: S5 Fig — The x-axis shows the enrichment score (-log10(pvalue)). The differentially expressed gene numbers in each term (gene group) are shown as the size of the bubbles. Each pvalue is also shown in color, as shown in the figure. (PDF) [file pone.0303260.s006.pdf]

S6 Fig

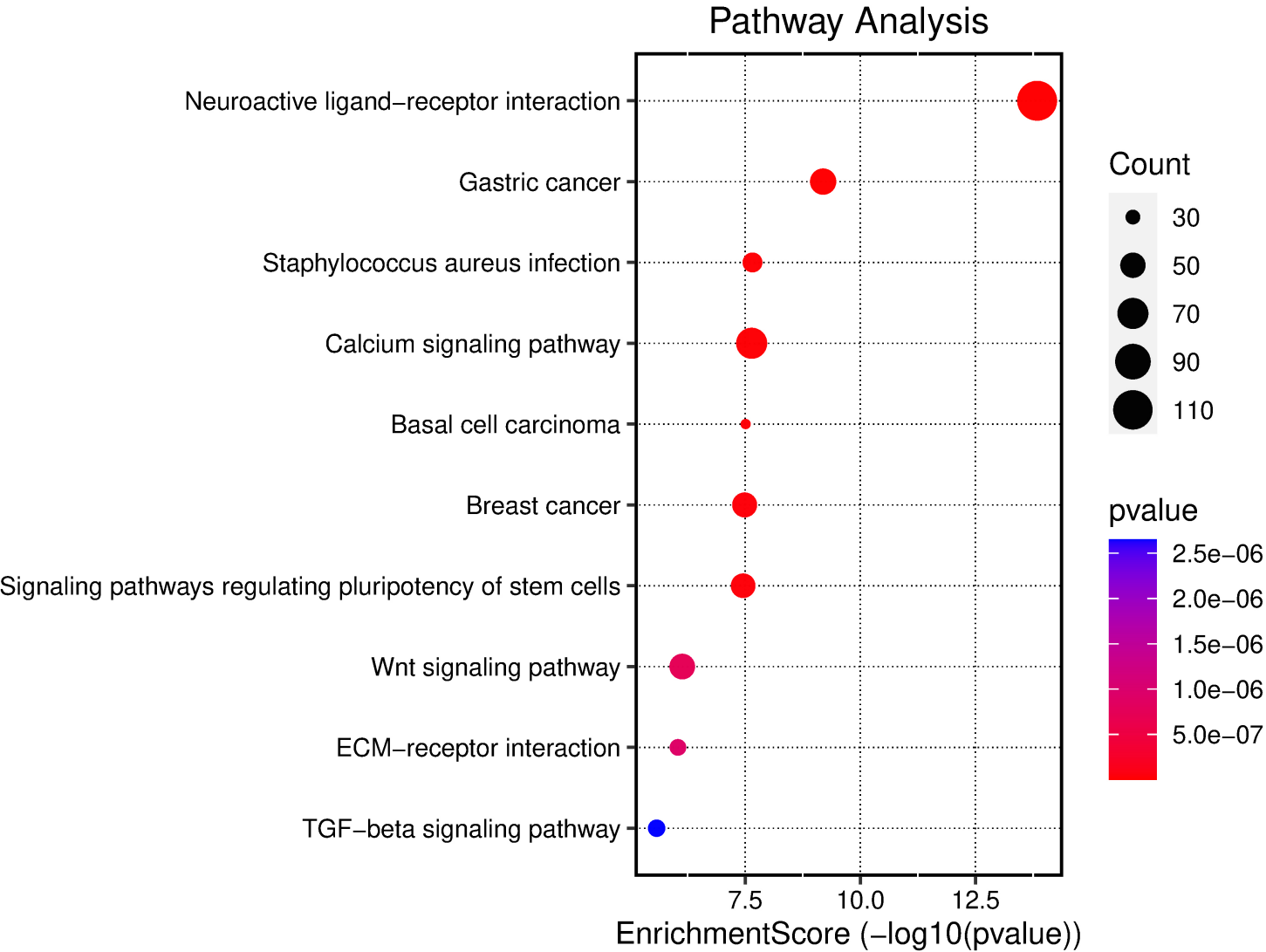

Supplement: S6 Fig — The gene expression changes were shown as the color of the gene-name box, as shown in the figure. (PDF) [file pone.0303260.s007.pdf]

S7 Fig

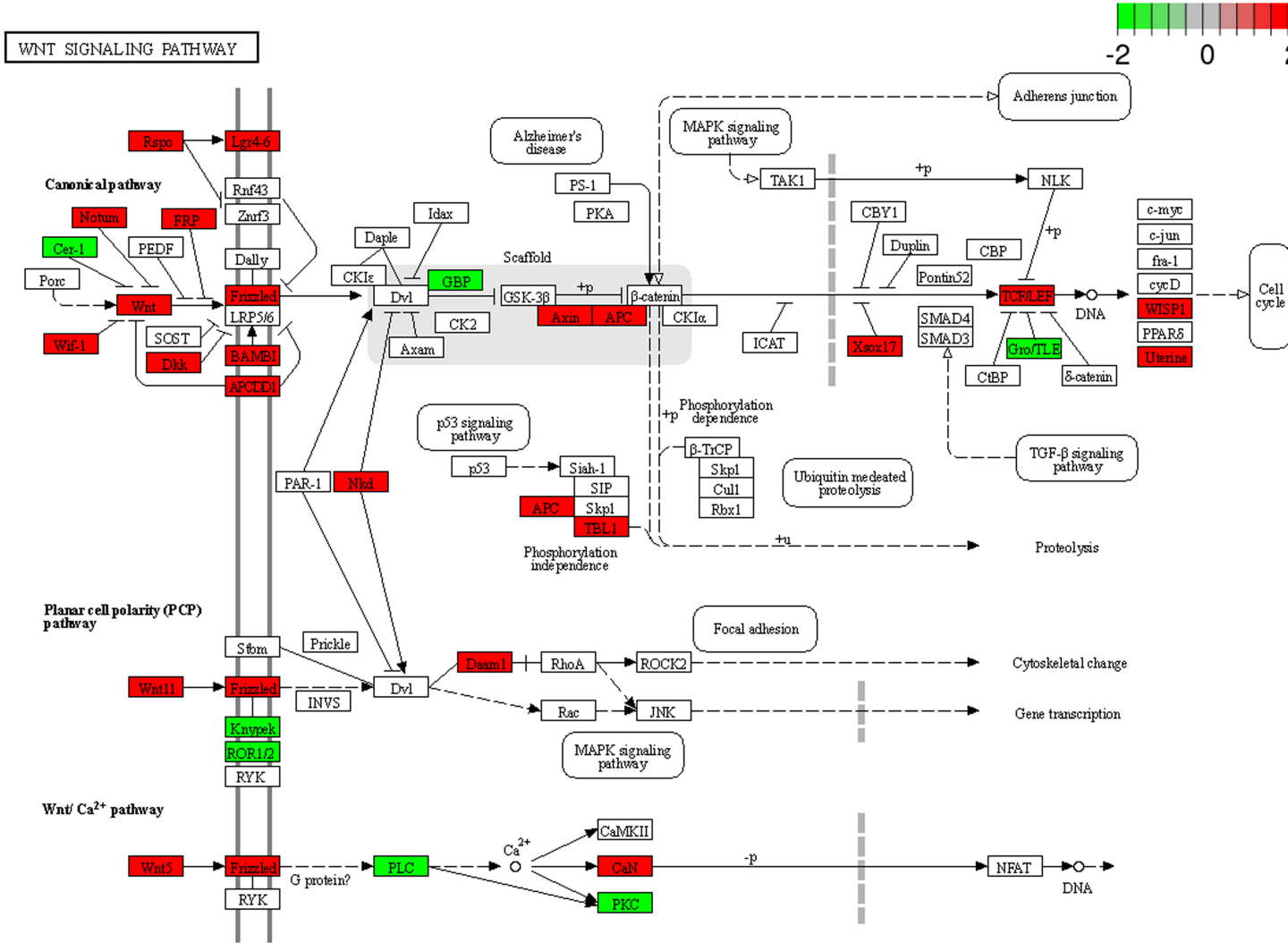

Data on KEGG graph  
Rendered by Pathview

Supplement: S7 Fig — The gene expression changes were shown as the color of the gene-name box, as shown in the figure. (PDF) [file pone.0303260.s008.pdf]

S8 Fig

A

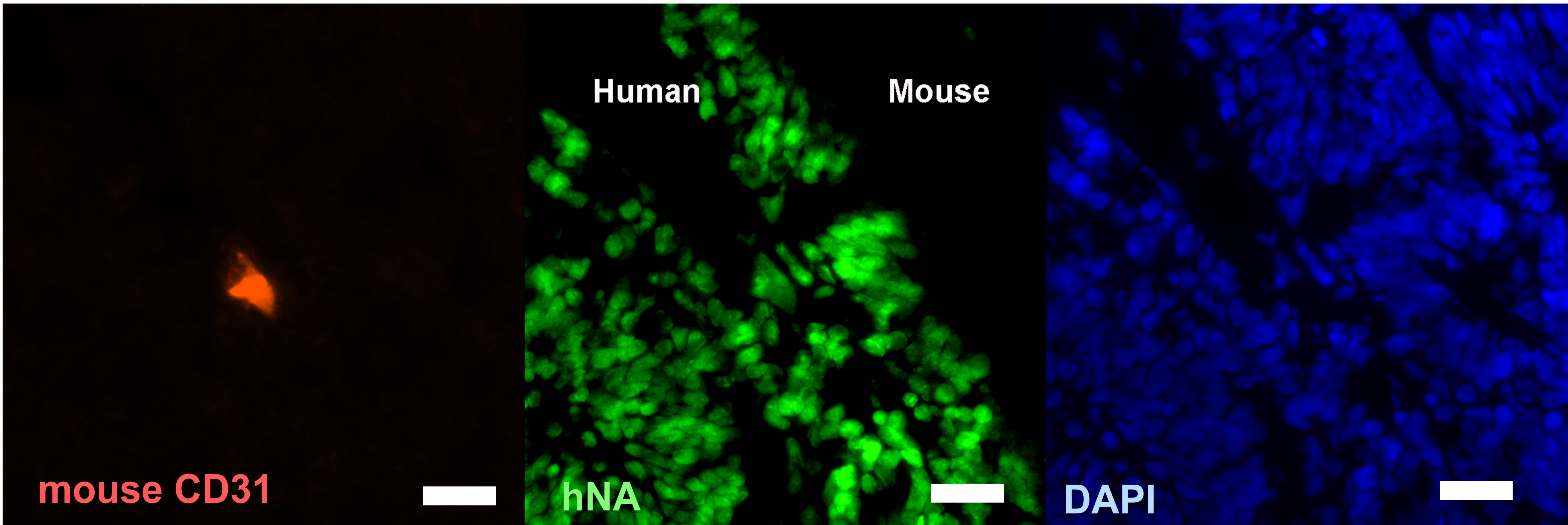

B

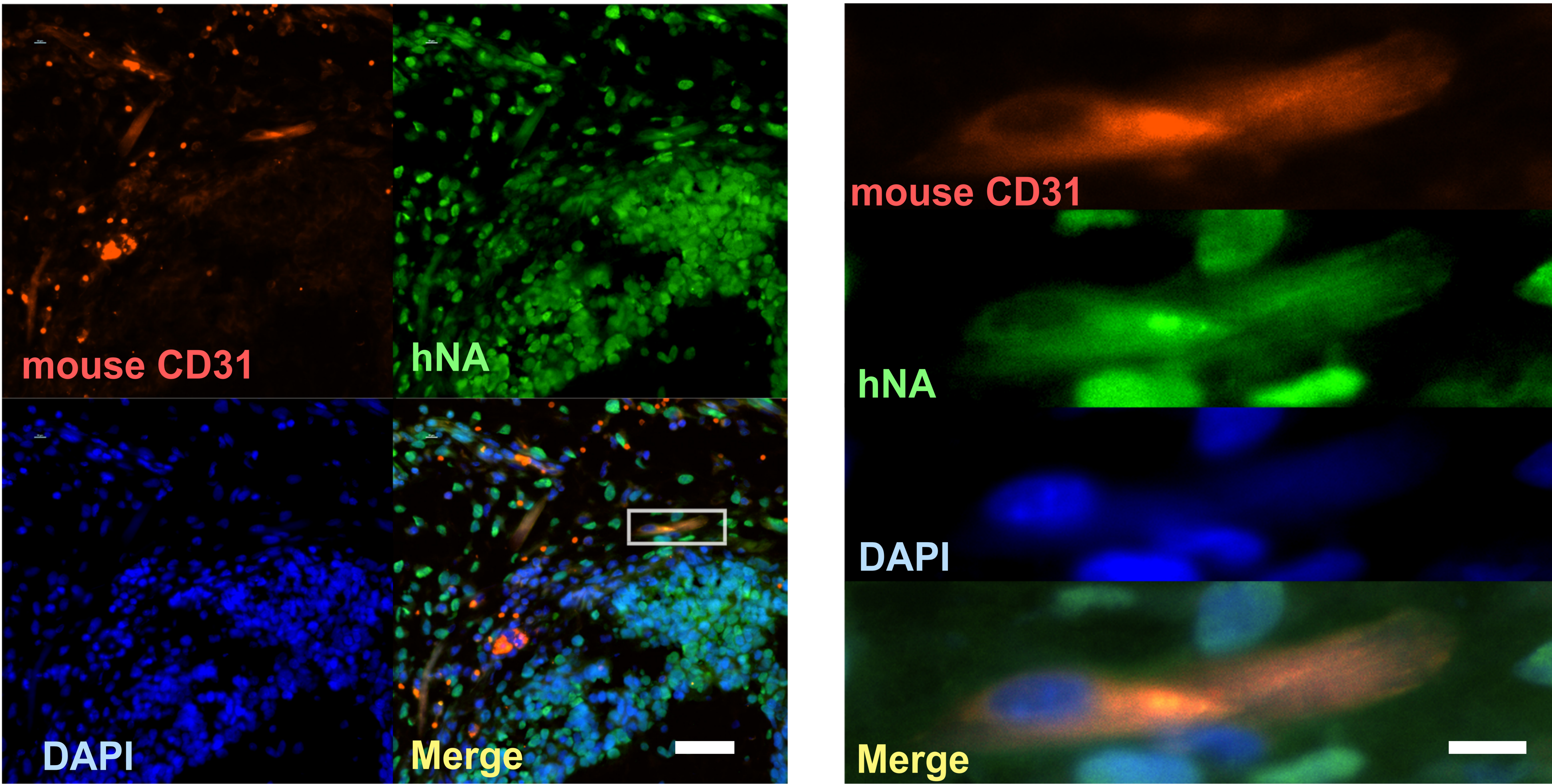

C

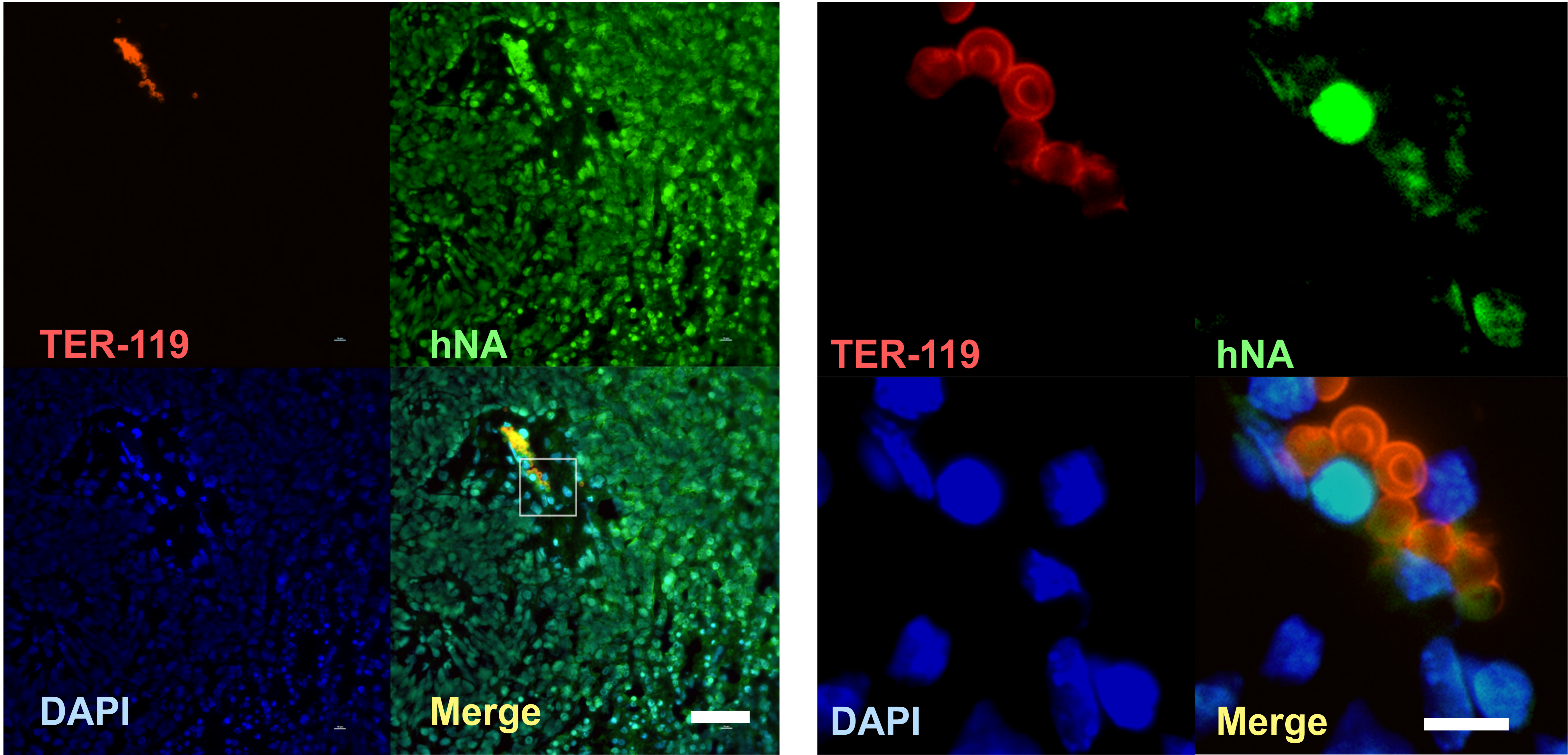

Supplement: S8 Fig — (A) Immunofluorescent staining focusing on the border zone between host- and the transplanted human-derived tissues in the lump tissue for mouse CD31 (red), human nuclear antigen (green) and all nuclear staining with DAPI (Blue). Scale bar = 20 μm (B) Immunofluorescent staining of the lump tissue-derived cryo-sections for mouse CD31 (red), human nuclear antigen (green). The right panel indicates the zoomed images of the white square-indicated area in the merged image. Scale bar = 50 μm (left), 10 μm (right). (C) Immunofluorescent staining for TER-119 (red) and human nuclear antigen (green). The right panel indicates the zoomed images of the white square-indicated area in the merged image. Scale bar = 50 μm (left), 10 μm (right). The used antibodies are shown in the S2 Table. (PDF) [file pone.0303260.s009.pdf]
