# Supplementary material for: Lgr6-expressing functional nail stem-like cells differentiated from human-induced pluripotent stem cells
Source: PLoS One. 2024 May 14;19(5):e0303260. doi: 10.1371/journal.pone.0303260 (PMC11093308; doi:10.1371/journal.pone.0303260)
Supplement: S2 Fig — Transcriptional regulatory factors (A), Morphological factors (B), and Keratins (C). The color strength of each column was assigned by the maximum-to-minimum numbers in each gene category. (PDF) [file pone.0303260.s003.pdf]

S2 Fig

A

GeneName	D0	D50
ZSCAN10	53.684	0.015
LINC00678	33.675	0.005
VRTN	65.595	0.015
NANOG	13.151	0.016
LIN28A	473.333	2.527
POU5F1	147.935	0.076
LCK	16.05	0
ESRG	206.047	0.048
IDO1	4.828	0.031
LGR5	0.089	2.38
LGR6	0.116	3.834
HOXA1	0	2.662
HOXA2	0	2.331
HOXA3	0	4.07
HOXA4	0	0.514
HOXA6	0	1.123
HOXA7	0	10.333
HOXA9	0	3.183
HOXA10	0	17.951
HOXA11	0	25.986
HOXA13	0	5.411
HOXB2	0	14.56
HOXB3	0	13.863
HOXB6	0	12.454
HOXB7	0	44.403
HOXB8	0	0.415
HOXB9	0	0.068
HOXB13	0	10.042
HOXC4	0	6.268
HOXC5	0	0.646
HOXC6	0	1.252
HOXC8	0	14.853
HOXC9	0	7.16
HOXC10	0	9.214
HOXC11	0	5.728
HOXC12	0	16.317
HOXC13	0.066	12.947
HOXD1	0	7.27
HOXD3	0	5.966
HOXD4	0	16.635
HOXD8	0	5.403
HOXD9	0	1.207
HOXD11	0	1.809
HOXD12	0	24.502
HOXD13	0	27.628

GeneName	D0	D50
TBX1	0.043	1.649
TBX2	0	7.207
TBX3	0.008	32.747
TBX4	0	0.514
TBX5	0.023	0.549
TBX15	0.129	1.988
TBX18	0.006	0.585
TBXT	0.039	1.814
DLX1	0.027	7.034
DLX2	0.052	4.015
DLX3	0.019	22.685
DLX4	0.055	6.15
DLX5	0	5.154
DLX6	0	7.337
PITX1	0	42.873
PITX2	0.107	3.116
EPHA2	8.939	49.458
EPHA3	0.04	17.314
EPHA4	0.666	34.816
EPHA5	0.026	1.437
EPHA7	1.242	4.844
EPHB1	1.177	7.727
EPHB3	0.252	10.717
SOX9	2.36	13.635
SOX10	0.596	2.471
SOX14	0.18	2.967
SOX17	0	0.402
SOX18	0	3.993
LMX1A	0	0.318
LMX1B	0.041	7.625
RUNX1	0.016	3.246
RUNX2	0.383	4.919
RUNX3	0.095	12.817
EN1	0	15.192

Max

Min

B

GeneName	D0	D50
BMP1	0.604	5.737
BMP2	0.863	25.058
BMP4	1.076	44.282
BMP5	0.013	7.715
BMP6	0.548	9.34
BMP7	2.117	37.147
GDF11	5.194	20.231
FGF3	0	14.89
FGF4	0.442	8.555
FGF7	0.016	0.213
FGF8	0.365	60.721
FGF9	0.11	15.496
FGF10	0	0.737
FGF14	0.004	0.077
FGF17	0.015	2.362
FGF18	0.674	40.497
FGF19	4.098	37.478
FGF20	0.047	7.09
TGFA	0.639	7.373
TGFB1	2.342	28.418
TGFB2	0.085	19.232
TGFB3	0.056	3.265
WNT1	0	0.252
WNT2	0	0.115
WNT3A	0.017	0.352
WNT4	0.056	3.842
WNT5A	0.378	57.838
WNT5B	0.346	5.404
WNT6	0	14.751
WNT7B	0.041	0.83
WNT10A	0	4.522
WNT10B	0	0.363
WNT11	0.138	4.74
WNT16	0.048	0.697
WLS	0.445	67.407
IGF1	0	0.67
IGF2	0	0.08
SHH	0.04	1.643
GLI3	1.691	11.069
NOG	0.135	13.545
GREM1	0.021	2.496
GREM2	0.025	1.754
VEGFD	0.047	6.247

C

GeneName	D0	D50
KRT1	0	12.02
KRT2	0	5.723
KRT5	0	22.326
KRT6A	0	129.8
KRT6B	0	24.441
KRT6C	0	45.059
KRT7	3.563	15.486
KRT9	0	0.338
KRT13	0	4.709
KRT14	0	7.332
KRT15	0.035	0.967
KRT16	0	142.643
KRT17	0.311	15.39
KRT18	137.734	45.578
KRT23	0	46.623
KRT24	0	0.324
KRT25	0	0.399
KRT27	0	1.11
KRT28	0	0.721
KRT31	0	49.011
KRT33A	0	1.32
KRT33B	0	10.473
KRT34	0	14.164
KRT36	0	2.945
KRT37	0	1.05
KRT38	0	5.423
KRT39	0	1.132
KRT71	0	4.389
KRT72	0	0.412
KRT73	0	0.335
KRT74	0	1.495
KRT75	0	0.537
KRT78	0	0.408
KRT80	0.107	4.831
KRT81	0.03	4.512
KRT82	0	0.797
KRT83	0	0.237
KRT84	0	12.253
KRT85	0	10.88
KRT86	0.086	6.936
