# Supplementary material for: Lgr6-expressing functional nail stem-like cells differentiated from human-induced pluripotent stem cells
Source: PLoS One. 2024 May 14;19(5):e0303260. doi: 10.1371/journal.pone.0303260 (PMC11093308; doi:10.1371/journal.pone.0303260)
Supplement: S1 Table — (PDF) [file pone.0303260.s010.pdf]

Table S1

Primers for qPCR

gene name		Sequence (from 5' to 3')
Hoxd13	Fw	AGGTGTACTGCACCAAGGACCA
	Rv	GCAGTTTGGTGTAAGGCACTCTC
Tbx5	Fw	AAATGAAACCCAGCATAGGAGCTGGC
	Rv	ACACTCAGCCTCACATCTTACCCT
Pitx1	Fw	GTACGCACTTCACAAGCCAGCA
	Rv	GCTCGGTGAGGTTGGTCCACA
Runx2	Fw	CCCAGTATGAGAGTAGGTGTCC
	Rv	GGGTAAGACTGGTCATAGGACC
Krt-17	Fw	GGTGGGTGGTGAGATCAATGT
	Rv	CGCGGTTCAGTTCCTCTGTC
Krt-81	Fw	GCATTGGGGCTGTGAATGTCT
	Rv	ACCCAGGGAGCTGATACCAC
Lgr5	Fw	GTTTCCCGCAAGACGTAACT
	Rv	CAGCGTCTTCACCTCCTACC
Lgr6	Fw	TGACGGCTTACCTGGACCTCA
	Rv	AGAGAATGCTTGTCCTGGGATG
Ribosomal protein 18s	Fw	GCGGCGGAAAATAGCCTTTG
	Rv	GATCACACGTTCCACCTCATC
