# Supplementary material for: Lgr6-expressing functional nail stem-like cells differentiated from human-induced pluripotent stem cells
Source: PLoS One. 2024 May 14;19(5):e0303260. doi: 10.1371/journal.pone.0303260 (PMC11093308; doi:10.1371/journal.pone.0303260)
Supplement: S2 Table — (PDF) [file pone.0303260.s011.pdf]

Table S2

The antibodies used in this study

Antigen Name	Host	Clonality	Manufacturer	I.D.	Dilution
Brachyury	Goat	polyclonal	Santacruz	SC-17743	1:100
E-cadherin	Rabbit	polyclonal	Santacruz	SC-7870	1:100
Hand2	Mouse	monoclonal	Santacruz	SC-398167	1:100
Pitx1	Mouse	monoclonal	Santacruz	SC-271435	1:100
Lgr6	Mouse	monoclonal	Santacruz	SC-393010	1:100
Keratin-17	Mouse	monoclonal	Santacruz	SC-393002	1:100
Keratin-81	Mouse	monoclonal	Santacruz	SC-100929	1:100
Keratin-6	Mouse	monoclonal	Santacruz	SC-514520	1:100
Keratin-16	Mouse	monoclonal	Santacruz	SC-53255	1:100
Keratin-31	Rabbit	polyclonal	ATLAS Antibodies	HPA049550	1:100
Human Nuclei	Mouse	monoclonal	Chemicon	MAB1281	1:100
GFP	Goat	polyclonal	Santacruz	SC-5385	1:100
TER-119 (Phycoerythrin conjugated)	Rat	monoclonal	Thermo Scientific	12-5921-82	1:100
Mouse CD31	Rat	monoclonal	BD	AB_393571	1:100

Product name	Host	Clonality	Manufacturer	I.D.	Dilution
rabbit IgG(H+L) AlexaFluor 488	Donkey	polyclonal	Thermo Scientific	A21206	1:200
rabbit IgG(H+L) AlexaFluor 546	Donkey	polyclonal	Thermo Scientific	A10040	1:200
goat IgG(H+L) AlexaFluor 488	Donkey	polyclonal	Thermo Scientific	A11055	1:200
goat IgG(H+L) AlexaFluor 546	Donkey	polyclonal	Thermo Scientific	A11056	1:200
mouse IgG(H+L) AlexaFluor 488	Donkey	polyclonal	Thermo Scientific	A21202	1:200
mouse IgG(H+L) AlexaFluor 546	Donkey	polyclonal	Thermo Scientific	A10036	1:200
rat IgG(H+L) AlexaFluor 594	Donkey	polyclonal	Thermo Scientific	A21209	1:200
